# Research progress on humoral biomarkers of Alzheimer’s disease: A review

**DOI:** 10.1097/MD.0000000000038978

**Published:** 2024-07-26

**Authors:** Hao Zhang, Xiaofeng Fu, Mengyu Yang, Xiaowen Song, Min Li, Xuezhen Wang

**Affiliations:** a Department of Neurology, Affiliated Hospital of Binzhou Medical College, Binzhou, China; b Binzhou Medical College, Binzhou, China; c Department of Ultrasound Medicine, Affiliated Hospital of Binzhou Medical College, Binzhou, China.

**Keywords:** Alzheimer’s disease, biomarkers, dementia, neurodegeneration

## Abstract

Alzheimer’s disease (AD) is a neurodegenerative disorder characterized by progressive memory loss. The main pathological features are neuronal fibrillary tangles caused by amyloid beta deposition and hyperphosphorylation of tau protein, accompanied by neuronal death and loss of synaptic structure. Early diagnosis is the key to the treatment of AD. It is known that some small molecular components are related to the pathogenesis of AD. This article will summarize the common AD biomarkers in cerebrospinal fluid and blood and analyze the current status of AD biomarkers and future research directions. This review summarizes the promising biomarkers for the diagnosis of AD in the last decade and describes their changes in AD body fluids. The diagnostic biomarkers related to AD were mainly distributed in cerebrospinal fluid and blood. Significant changes in these molecules can be detected in cerebrospinal fluid and blood, and they are correlated with AD severity. These humoral molecules have necessary relationship with AD and can be used as AD biomarkers to assist early diagnosis of AD.

## 1. Introduction

Alzheimer’s disease (AD) is a neurological disease characterized by slow forgetting. AD is considered to be the most common form of Senile dementia and consists of 2 main forms, a rare early-onset dementia, which usually occurs before age 65, and a late-onset dementia, which occurs after age 65 and is the most common form.^[1]^ AD is mainly characterized by cognitive decline, including forgetfulness, loss of judgment, mood and personality changes, and finally complete loss of self-cognition, thereby cutting off contact with the outside world. It is the most fatal neurodegenerative disease in the world.^[2]^ Amyloid beta deposition and neuronal tangles are the main features of AD, brain immune system disorder and synaptic structure damage are also considered to be important components of the pathogenesis of AD.^[3,4]^ These changes begin 15 to 25 years before the onset of cognitive symptoms.^[5]^ At present, the number of AD patients is increasing year by year in the global situation, which poses a serious challenge to the world public health cause.^[6]^ Demographics also showed that there were individual differences in the pathogenesis and pathological manifestations of AD individuals.^[7]^ Therefore, more effective biomarkers are needed for early diagnosis of AD in the absence of cognitive changes.

With the continuous exploration of the pathogenesis of AD, on the basis of the pathogenesis, there are many proteins and cytokines that are specifically changed in the body fluids of AD patients. These specific changes of cellular molecules were consistent with the degree of AD damage to a certain extent. In recent years, clinical marker tests have been carried out,^[8]^ and some of these molecules have been recognized by the public as potential biomarkers of AD. These molecules are detected in the body fluids of patients and change with AD. Common biomarkers of AD in body fluids, including cerebrospinal fluid and blood, will be summarized below and classified according to the pathway of action.

## 2. Biomarkers in cerebrospinal fluid

### 2.1. Amyloid beta (Aβ)

The beta-amyloid mechanism has been recognized as the main culprit leading to AD, which runs through the entire pathogenesis of AD.^[9]^ It is found that the level of Aβ-42 in the cerebrospinal fluid of AD patients is significantly down-regulated, which is caused by the excessive precipitation of Aβ in the brain tissue, which leads to the gradual reduction of free Aβ in the cerebrospinal fluid.^[10]^ Palmqvist et al, found that Aβ in cerebrospinal fluid decreased even before irreversible deposition of Aβ in AD.^[11]^ In the absence of relevant diagnostic evidence in the early stage of AD, the determination of Aβ in cerebrospinal fluid has been recognized as A method with high accuracy.^[12]^

### 2.2. Tau

Tau is a microtubule-binding axon protein widely expressed in neurons. It has a complex structure, and its main function is to promote microtubule assembly and stability.^[13]^ Tau, as another major pathological change in Alzheimer’s disease, has significant changes in AD cerebrospinal fluid. Tau exerts neurotoxicity in AD mainly by its own excessive phosphorylation and abnormal aggregation, forming neurofibrillary tangles.^[14]^ In addition to its function within neurons, Tau is secreted into brain interstitial fluid that freely communicates with the cerebrospinal fluid.^[15]^ Through the detection of specific Enzyme Linked Immunosorbent Assay system, it was found that the stability of total tau protein (T-tau) and phosphorylated tau protein (P-tau) increased in AD cerebrospinal fluid.^[16]^ The measurement of tau in cerebrospinal fluid can directly reflect the degree of neuron and synapse damage.^[17]^

### 2.3. Markers related to loss of synaptic function

#### 2.3.1. Neurogranin

Neurogranin is a postsynaptic protein composed of 78 amino acids, which is widely expressed in the central nervous system and plays an important role in synaptic function.^[18]^ Neurogranin has been shown to affect synaptic plasticity, synaptic regeneration and long-term enhancement mainly through calcium and calmodulin signaling pathways.^[19]^ As a postsynaptic protein, the content of neurogranin in cerebrospinal fluid has become an effective biomarker of synaptic loss or dysfunction.^[20]^ Agnello et al, showed that the expression level of neurogranin in the cerebrospinal fluid of AD patients was significantly higher than that of non-AD patients.^[21]^ In AD, neurogranin is significantly associated with total tau and phosphorylated tau and cognitive decline.^[22]^ As a biomarker reflecting the integrity of synapses in the brain, neurogranin has obvious consistency with AD, which can provide effective evidence for the early diagnosis of AD.^[23]^

#### 2.3.2. α-Synuclein

α-Synuclein is a small protein composed of more than 100 amino acid residues. The hydrophilic and lipophilic N-terminus (amino acids 1–60), hydrophobic core region (amino acids 61–95) and unstructured region together constitute the primary structure of the protein.^[24]^ The hydrophobic core region of structure plays a major role in the aggregation and folding of α-synuclein. At present, α-synuclein has been found to be correlated with a variety of neurodegenerative diseases, and has different expression in different diseases.^[25]^ Garcia et al found a significant positive correlation between alpha-synuclein and tau in cerebrospinal fluid of AD patients, and both showed similar changes in AD episodes, which was also confirmed in animal experiments.^[26]^ Consistent with the changes in tau, α-synuclein was significantly upregulated in cerebrospinal fluid of AD patients.^[21]^ The neurotoxicity of α-synuclein mainly lies in the alteration of synaptic function. In previous studies, it was also believed that the decrease of synaptic vesicle protein and the change of synaptic vesicle protein composition were the key factors for α-synuclein to cause AD.^[26,27].^At present, the quantitative analysis of α-synuclein in cerebrospinal fluid is mainly achieved by αsyn Pheromone-based Multi-Mobile-Robots Collaborative Control Algorithm and αsyn Real Time-Quaking Induced Conversion, which can further shorten the detection time under the premise of ensuring the specificity. It is helpful to detect the changes of α-synuclein in the early stage of AD.^[28]^

#### 2.3.3. Synaptosomal associated protein-25

Synaptosomal associated protein-25 (SNAP-25) is a synaptosome associated protein that is visible in the human hippocampus and plays a role in the transmission of synaptic structural neurotransmitters. The study of Sutphen et al, found that the expression of SNAP-25 was decreased in the brain tissue of AD patients, but it was highly expressed in the cerebrospinal fluid, and was highly positively correlated with the injury degree of AD.^[29]^ This phenomenon exists in the early stage of AD and gradually progresses with the prolongation of the disease course.

#### 2.3.4. Growth-associated protein-43

Growth-associated protein-43 (GAP-43) is a growth-related presynaptic protein that is highly expressed in the human hippocampus and is known to play an important role in the regulation of axonal growth, synaptic plasticity, learning, and memory.^[30]^ Previous studies have found that GAP-43 is closely related to AD and is involved in the pathophysiology of AD. The Enzyme Linked Immunosorbent Assay technology was used to detect GAP-43 in human cerebrospinal fluid, and GAP-43 showed a high level of expression in the cerebrospinal fluid of AD patients, which was consistent with the changes of T-tau and P-tau.^[31]^ Compared with other neurodegenerative diseases, GAP-43 is more obvious in the cerebrospinal fluid of AD, and it is one of the specific biomarkers of AD.

### 2.4. Biomarkers related to neuroinflammation

#### 2.4.1. YKL-40

Ykl-40, an astrocyte protein encoded primarily by the gene Chi3l1, is a well-known biomarker of neuroinflammation in human cerebrospinal fluid. Its expression increased with the course of AD.^[32]^ Ykl-40 is expressed in both astrocytes and microglia, and the specific mechanism leading to AD is still unclear.^[33]^ During the preclinical, prodromal, and dementia stages of AD, YKL-40 levels in cerebrospinal fluid are elevated, accompanied by elevated T-tau and P-tau protein levels, especially in individual patients with significant Aβ deposits. Higher levels of YKL 40 increase the risk of progression to dementia in non-dementia patients.^[34]^

#### 2.4.2. Triggering receptor expressed on myeloid cells 2

Triggering Receptor Expressed On Myeloid Cells 2 (TREM2) is a 230-amino acid cell-surface transmembrane glycoprotein encoded by the TREM2 gene on chromosome 6p21. TREM2 can participate in neuroinflammation and play a role in AD by mediating the activation and phagocytosis of microglia.^[35]^ Trem-2 is expressed in microglia in the brain, and its extracellular domain is cleaved by proteases and secreted out of the cells, allowing it to be detected in the cerebrospinal fluid.^[36]^ At present, it is found that the expression level of STREM-2 in the cerebrospinal fluid of AD patients is significantly higher than that of healthy people, and the change trend is consistent with that of tau, and there is an obvious positive correlation.^[37]^

#### 2.4.3. Astrocytes

In the early stages of AD, we observed A significant increase in cerebrospinal fluid astrocytes, and these changes occurred even before Aβ deposition.^[38–40]^ Astrocytes are involved in A variety of neural functions, including neuronal nutritional support, neurotransmitter transmission and recycling, CBF regulation, and Aβ clearance and degradation. All these processes are obviously dysregulated in AD.^[41]^It can be seen that astrocytes play different roles in different stages of AD. Reactive astrocytes release reactive oxygen species and nitrogen, tumor necrosis factor-α, matrix metalloproteinase-9 and other cytokines, which have direct neurotoxicity.^[42]^ There is a growing consensus that glial cell-mediated inflammation is a major contributor to the degenerative process and cognitive loss in AD.^[43]^ Astrocytes and their released cytokines can be used as new biomarkers for AD and provide new targets for AD treatment.

### 2.5. MicroRNA

In addition to genetic variation, epigenetics also plays an important role in the pathogenesis of AD, including the influence of MicroRNA on AD.^[44]^ Through the analysis and detection of peripheral blood, exosomes, cerebrospinal fluid and extracellular fluid of AD patients, it was found that a variety of micrornas were obviously dysregulated.^[44]^ In animal experimental models, miR-331-3p and miR-9-5p were significantly up-regulated in the cerebrospinal fluid of AD, and they could participate in the process of autophagy, promote the deposition of amyloid protein, and aggravate the degree of cognitive impairment in AD.^[45]^ MiR-22-3p and miR-126-3p were significantly down-regulated in AD. After increasing the expression of miR-22-3p and miR-126-3p, cognitive impairment was significantly improved, which may be related to the neuroinflammatory mechanism.^[46,47]^ With the continuous progress of research, it was found that the expression of miR-107, miR-26b, miR-30e, miR-34a, miR-485, miR-200c, miR-210, miR-146a, miR-34c, miR-125b and other micrornas in AD also showed differences.^[48]^ During the development of AD, MicroRNA can not only regulate the plasticity of neural synapses and affect the homeostasis of neurotransmitters, but also promote the release of related inflammatory factors to induce neuroinflammation and accelerate or slow down the progression of AD.^[49,50]^ Interestingly, MicroRNA dysregulation can be detected in the cerebrospinal fluid of patients in the early stage of AD disease, which is one of the new biomarkers of AD in the future.

## 3. Biomarkers in blood

### 3.1. Amyloid beta (Aβ)

As the main pathological manifestation of Alzheimer’s disease, Aβ deposition in the brain and the related inflammatory response triggered by Aβ are believed to be an earlier event before neuronal loss.^[51]^ Since Aβ in blood and brain have different sources, the relationship between Aβ in blood and AD has not been clarified.^[52]^ In the study of Nakamura et al, Aβ in plasma was measured and analyzed by immunoprecipitation combined with mass spectrometry, and it was confirmed that there was a significant correlation between the change of Aβ content in blood and that in cerebrospinal fluid.^[53]^ Sun et al, confirmed through bone marrow cell transplantation in mice that the continuous increase of Aβ in the blood can lead to increased deposition of Aβ in the brain, and eventually lead to significant cognitive decline. When Aβ in the blood is reduced, cognitive impairment can be improved, which indicates that Aβ in the blood is closely related to Aβ in the brain.^[54]^ The changes of Aβ in blood can indirectly reflect the deposition of Aβ in brain and become potential biomarkers in AD blood.

### 3.2. Tau

The source of tau in blood includes 2 ways: peripheral source and brain source. Brain-derived tau can enter the bloodstream via the neuronal exosome pathway, which seems to be an easy process.^[55]^ A systematic analysis of previous studies revealed a clear association between plasma T-tau levels and Alzheimer’s disease.^[52]^ Tang et al performed cognitive grading on more than 3000 volunteers by Mini-Mentalstateexamination scale and collected blood samples from them to measure Tau protein, and found that plasma Tau protein was significantly negatively correlated with Mini-Mentalstateexamination score.^[56]^ This suggests that when plasma tau is increased, the degree of cognitive impairment is aggravated. In addition, after combing and analyzing the role of tau in AD, Rik et al concluded that the expression of plasma p-tau combined with brief cognitive tests and apolipoprotein genotyping could more accurately predict the early individual development of AD, and also emphasized the key role of blood p-tau in the pathogenesis of AD.^[57]^ All of this suggests that tau in the blood, p-tau is a key biomarker for early identification of AD.

### 3.3. Neurogranin

With more and more studies on neurogranin in cerebrospinal fluid. Research on neurogranxin is gradually moving into the blood. Liu et al found that neurogranin in blood neurogenic exosomes of AD patients showed a significant decrease compared with healthy people of the same age. There was no significant change in plasma granuloprotein. This may be related to the excessive breakdown of neurogranin in exosomes and brain tissue into disulfide bridge or glutathione modified peptides and their release into the cerebrospinal fluid.^[58]^ This is the exact opposite of the changes in the cerebrospinal fluid, where neurogranin in the blood neurogenic exosomes has changed even 5 to 7 years before the onset of cognitive impairment.^[59]^ It may be more sensitive to the diagnosis of AD at the stage of mild cognitive impairment. With the enhancement of the detection technology of blood neurogenic exosomes, neurogranin in blood neurogenic exosomes will become an effective biomarker for the early diagnosis of AD.

### 3.4. MicroRNA

MicroRNA is not only dysregulated in cerebrospinal fluid of AD patients, but also has similar changes in peripheral blood.^[60]^ Cross analysis of dysregulated miRNAs in peripheral blood of AD patients and miRNAs found in the brain vulnerable region revealed 10 microRNAs, including miR107, miR26b, miR30e, miR34a, miR485, miR200c, miR210, miR146a, miR34c, miR125b in the early stage of AD peripheral blood dysregulation.^[48]^ MiR125b, miR146a, miR200c, miR26b, miR30e, miR34a, and miR34c showed an up-regulated trend, while miR107, miR210 and miR485 showed a downward trend.^[61]^ This indicates that there may be a non-negligible association between miRNAs in the blood and AD. MiRNAs are mainly present in plasma and exosome vesicles in blood, where the miRNAs in exosome vesicles exhibit more stable expression in blood and are the main targets for detecting AD-related miRNA expression in blood.^[62]^ With current technology applications based on chips and biosensors, detection of miRNAs in blood is a more minimally invasive method than brain tissue biopsy and cerebrospinal fluid analysis.^[63]^

## 4. Conclusion

As a worldwide public health problem, AD seriously damages the quality of human life. Early treatment is the key measure to effectively improve the damage of AD. The onset of AD is insidious, and early diagnosis is very important. The current definitive diagnosis of AD is the observation of Aβ plaques and neurofibrillary tangles in brain tissue.^[64]^ This is not feasible in reality. Therefore, effective humoral biomarkers are necessary for the early diagnosis of AD. At present, the detected humoral markers mainly exist in cerebrospinal fluid and blood, and are closely related to the pathogenesis of AD. As for the biomarkers in cerebrospinal fluid, amyloid beta hypothesis and tau hypothesis are the commonly accepted characteristic pathological hypotheses of AD. The combination of extracellular deposition of Aβ and intracellular hyperphosphorylation of tau leads to AD.^[65]^ They are expressed in brain tissue and can also be detected in cerebrospinal fluid. They are the most representative biomarkers of AD. In addition, the occurrence of AD is inseparable from neuroinflammation and loss of synapses. Indicators of neuroinflammation, including YKL-40, sTREM2, astrocytes, and molecules related to synaptic structure and function, including neurogranin, α-synuclein, SNAP-25, GAP-43, are also obviously dysregulated in cerebrospinal fluid. Most of them have obvious correlation with tau changes. These molecules are also closely related to the degree of AD injury and are potential biomarkers of AD in cerebrospinal fluid. Due to the existence of the blood-brain barrier, there are few biomarkers in the blood for the detection of AD at present. In addition to AD specific markers such as Aβ and tau, neurogranin has significant changes in the blood of AD, showing A significant negative correlation with the degree of cognition, which provides more evidence for the early diagnosis of AD. MicroRNAs have long been in the public eye as gene-level biomarkers. Whether in cerebrospinal fluid or blood, there are a variety of MicroRNA and AD have obvious correlation. MicroRNA may act on AD through a variety of complex pathways. MicroRNAs are regarded as potential biomarkers and therapeutic agents for AD, and they are proposed as potential therapeutic targets for the disease.^[66]^ It provides a new direction for the study of AD gene level.

The traditional method of blood collection, transportation, and storage is not only time-consuming and labor-intensive but also significantly impacts the stability of blood samples. With advancements in science and technology, new techniques have been implemented. The noninvasive blood collection technology represents a novel approach to reforming traditional blood collection methods, utilizing specific spectral changes to detect target proteins and genes. The efficacy of noninvasive blood collection techniques has been validated in multiple studies and is comparable to the results obtained from invasive tests.^[67,68]^ The incorporation of pneumatic logistics with advanced robotics represents a novel technological advancement in the transportation of medical drugs and samples. The integration of pneumatic logistics with intelligent robotics offers higher efficiency and greater sample stability compared to traditional transportation modes, thereby enhancing operational efficiency.^[69]^

At present, AD is still an incurable disease with complex pathogenic pathways, and the relationship between various pathways is still unclear. At present, the research on AD markers is still in progress, and there are still no accurate and feasible biomarkers to be used. With the expansion of the range of AD biomarkers, investigating AD phenotypic heterogeneity has emerged as a promising approach for identifying effective markers, and may hold the key to advancing neuropathology and clinical radiology in the study of Alzheimer’s disease.^[70]^ Novel discoveries, such as epigenetic changes and the identification of aging measures, are also emerging as effective markers for AD, thus expanding the scope of potential AD markers.^[71]^ The screening of AD biomarkers still needs further in-depth study and more theoretical and practical basis.

## Acknowledgments

We thank Shandong Provincial Natural Science Foundation for the financial support of this experiment. We would like to thank the Institute of Metabolism, Binzhou Medical College for providing the experimental site and technical support. We thank the reviewers who reviewed this article and the MJEditor (www.mjeditor.com) for editing the English of this manuscript.

## Author contributions

**Conceptualization:** Mengyu Yang.

**Data curation:** Min Li.

**Formal analysis:** Xiaowen Song.

**Project administration:** Xiaofeng Fu.

**Writing – original draft:** Hao Zhang.

**Writing – review & editing:** Xiaofeng Fu.
